# Three-dimensional spectral domain optical coherence tomography and light microscopy of an intravitreal parasite

**DOI:** 10.1186/s12348-015-0064-x

**Published:** 2015-11-19

**Authors:** Aziz A. Khanifar, Michael J. Espiritu, Jane S. Myung, Grant D. Aaker, Audrey N. Schuetz, Donald J. D’Amico, R. V. Paul Chan

**Affiliations:** Department of Ophthalmology, Weill Cornell Medical College, New York, NY USA; Department of Pathology and Laboratory Medicine, Weill Cornell Medical College, New York, NY USA; Department of Ophthalmology and Visual Sciences, Illinois Eye and Ear Infirmary, University of Illinois at Chicago, Chicago, IL USA

**Keywords:** Gnathostomiasis, *Gnathostoma*, Ophthalmomyiasis, Optical coherence tomography, Parasitic infection, Retinal imaging

## Abstract

**Background:**

Various imaging modalities play a role in diagnosing parasitic infections of the eye. We describe the spectral domain optical coherence tomography (SD-OCT) findings of an intravitreal parasite with subsequent evaluation by light microscopy.

**Findings:**

This is a case report of a 37-year-old Ecuadorian man who presented with uveitic glaucoma and a new floater in his left eye for 1 week’s duration. Full ophthalmic examination revealed an intravitreal parasite. Color fundus photography, fluorescein angiography (FA), ocular ultrasonography (US), and SD-OCT were performed. The parasite was removed via 23-gauge pars plana vitrectomy and sent to pathology for evaluation.

Color fundus photography and ocular ultrasonography demonstrated an elongated foreign body within the vitreous above the retina. FA demonstrated minimal vascular changes in the vicinity of the parasite. SD-OCT was utilized to visualize the parasite and to create a three-dimensional (3D) image. The parasite was determined to be most consistent with *Gnathostoma* spp. by morphologic analysis.

**Conclusions:**

This is the first reported case of SD-OCT of an intravitreal parasite with corresponding evaluation by pathology. SD-OCT allows non-invasive, high-resolution visualization and 3D reconstruction of parasitic anatomy which may help establish tomographic criteria for species identification.

**Electronic supplementary material:**

The online version of this article (doi:10.1186/s12348-015-0064-x) contains supplementary material, which is available to authorized users.

## Findings

### Introduction

Intraocular parasites are uncommon occurrences in the USA. Diagnosis is dependent on reliable history of ingested foods, geographic location, season, and direct visualization of the parasite with ophthalmoscopy. Various imaging modalities have also been applied in the study of intraocular parasites such as fluorescein angiography. Ultrasound biomicroscopy has been used to localize larvae when not seen on dilated fundus examination [1], and scanning electron microscopy has aided morphological study for species determination [2, 3].

While optical coherence tomography (OCT) has been utilized in visualizing tracks of nonspecific cutaneous larva migrans through the epidermis [4], its capabilities have yet to be explored with regard to complete visualization of an intraocular helminthic infection. We report a case in which spectral domain optical coherence tomography (SD-OCT) imaging of an intravitreal parasite was performed. The clinical presentation and pathological findings are most consistent with *Gnathostoma* spp.

### Case report

A 37-year-old Ecuadorian immigrant presented to the New York-Presbyterian Hospital Emergency Department with a 3-day history of redness, pain, and hazy vision in his left eye. He denied flashes, photophobia, or discharge in either eye. He had no complaints in his right eye.

Two weeks prior to presentation, he experienced fevers, nasal congestion, and muscle aches, which spontaneously resolved. He also admitted to recently seeing a curved floater in his left eye. His past medical history was significant only for Bell’s palsy on the left in 2004, which had resolved spontaneously without ocular complications. His last tuberculin skin test was 3 years prior to presentation and was negative. He denied any risk factors for HIV. His past social history revealed that he immigrated to the USA from Ecuador 13 years ago and has not been back to Ecuador for 9 years. He denied any other travel, farm work, or extended contact with animals. The patient worked in a local restaurant as a cook, handling raw beef, pork, poultry, and both saltwater and freshwater fish with his bare hands. He reported that his duties include preparing the tuna tartar, and he usually eats any leftover food.

**Fig. 1:**
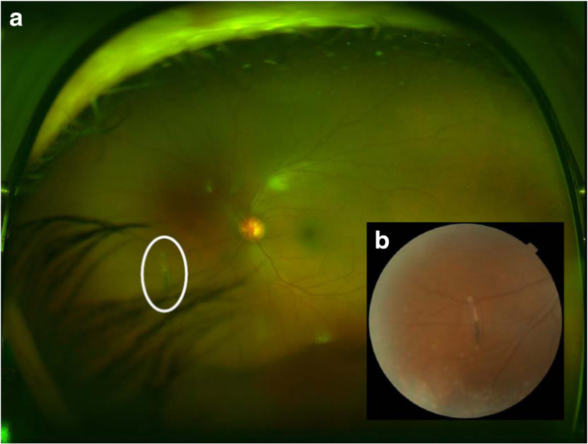
**a** Wide-angle color fundus photograph of the patient’s left eye demonstrating the parasite (*white oval*) within the vitreous and nasal to the optic disc. **b** Higher magnification color photograph of the intravitreal parasite

**Fig. 2:**
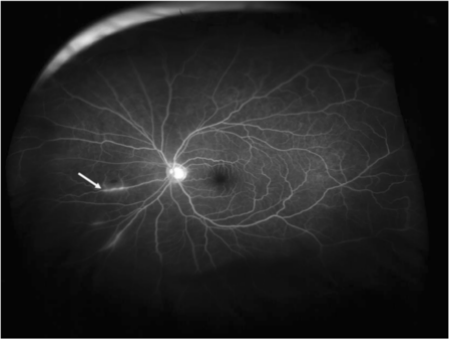
Wide-angle fluorescein angiogram demonstrating vascular sheathing (*white arrow*) in the region where the parasite was initially visualized

**Fig. 3:**
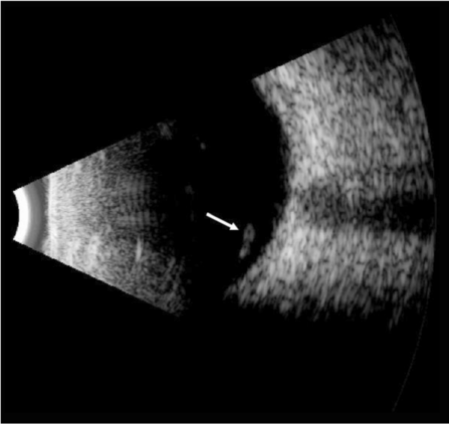
Ocular ultrasonography demonstrating the parasite (*white arrow*) within the vitreous. Note that the parasite is not in contact with the retina

**Fig. 4:**
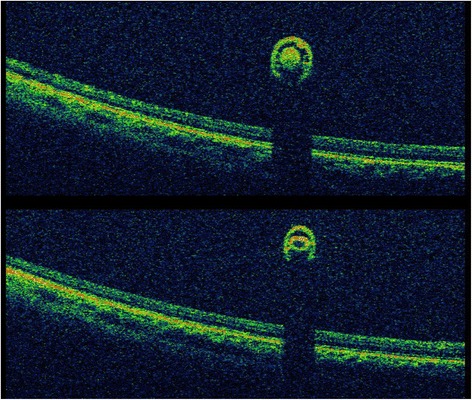
Spectral domain optical coherence tomography (SD-OCT) images of the parasite demonstrating *two concentric circles* within the vitreous above the retina. Note that the *inner circle* is hyper-reflective superiorly (**top image**) and hypo-reflective inferiorly (**bottom image**)

**Fig. 5:**
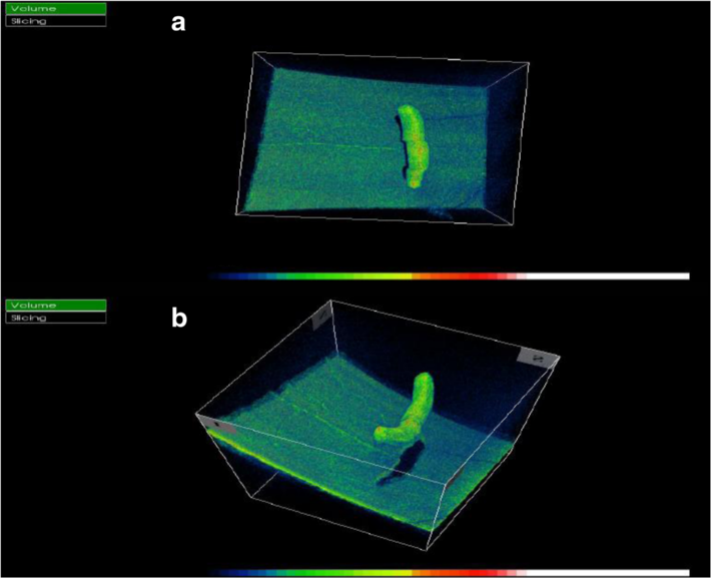
**a** Three-dimensional reconstruction of spectral domain optical coherence tomography (SD-OCT) images of the intravitreal parasite. **b** Additional view of SD-OCT image from different angles

On examination, his visual acuity was 20/20 in both eyes with pinhole. Intraocular pressure (IOP) was 12 and 35 mm Hg in his right and left eyes, respectively. Pupils, motility, confrontational visual fields, and external exam were within normal limits bilaterally. Slit lamp biomicroscopy and dilated fundus examination were normal for the right eye. In the left eye, the conjunctiva and sclera were quiet, and the cornea had microcystic edema and fine keratic precipitates. Cells were present in the anterior chamber, but there was no hypopyon. The iris and lens were normal. Dilated fundus exam revealed a peripheral operculated hole in the left eye and a single linear object measuring approximately 3 mm in length floating in the nasal vitreous. The object appeared to have a white disc followed by a whitening of the body on one end and a white pointed tip preceded by a reddish brown coloration of the body at the other end (Fig. 1). No vitritis, papillitis, vasculitis, retinitis, and other retinal breaks or detachments were noted. Ultra-widefield fluorescein angiography demonstrated mild vascular sheathing in the region where the parasite was originally seen (Fig. 2). Ocular ultrasonography verified that the object was not in contact with the retinal surface (Fig. 3). Using the Topcon 3D OCT-1000 (Topcon Medical Systems, Paramus, NJ), the intravitreal object was imaged (Fig. 4a, b and Additional file 1: Video 1). Three-dimensional (3D) reconstructions using the OCT B-scans were also created (Fig. 5a, b and Additional file 2: Video 2). Review of the scans demonstrated a tubular structure with a central lumen overlying the nasal retina. Superiorly, the lumen contained hyper-reflective material (Fig. 4a), whereas the inferior lumen was hypo-reflective (Fig. 4b).

Further diagnostic evaluation of the patient revealed peripheral eosinophilia, and magnetic resonance imaging of the brain was negative for cerebral larvae. Based on its appearance, gnathostomiasis was high on the differential diagnosis. A 23-gauge pars plana vitrectomy with removal of the foreign body was performed. Intraoperatively, the presumed worm was noted to be associated with superficial retinal hemorrhages and resist aspiration. The worm was eventually aspirated with a vitreous cutter using aspiration only. The specimen, within the tip of the vitreous cutter, was sent for microscopic evaluation by the Department of Pathology and Laboratory Medicine at New York-Presbyterian Hospital. The patient was discharged on oral ivermectin and recovered well.

**Fig. 6:**
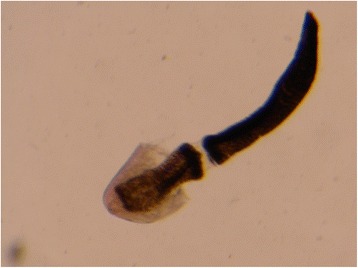
Light microscopy photograph of the parasite removed from the patient’s vitreous cavity. The cuticle has been partially removed

**Fig. 7:**
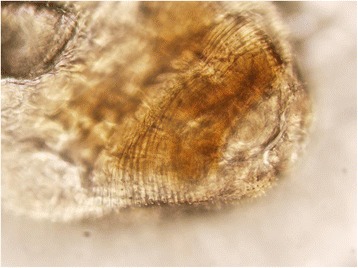
Higher magnification photograph of the rostral portion of the parasite demonstrating minute cuticular spines encircling the cuticular armature of the head bulb

Macroscopically, the single larva was light red-brown in color and 3 mm in length when viewed under a dissecting microscope (Fig. 6). Only a portion of the worm was available for examination after removal from the vitreous cutter, with a portion of the cuticle removed. Through examination of the remaining worm, including the probable gastrointestinal tract and the remnant of the anterior portion of the worm, a differential diagnosis was generated. Based on the size of the larva and the minute spines present on the cuticular armature of the head bulb on the anterior portion of the worm (Fig. 7), *Gnathostoma* spp. was the most likely diagnosis. The differential included bot fly larva or *Thelazia* spp. based on ocular localization, but the organism shape and size were not consistent with those organisms.

### Discussion

To our knowledge, this is the first reported case of intravitreal 3D SD-OCT imaging of a parasite in vivo and subsequent evaluation with light microscopy. Other investigators have utilized OCT to follow complications of parasitic infestation, but imaging of the actual parasite has rarely been reported [5–11].

Gnathostomiasis is a rare parasitic infection that can affect multiple organ systems, including the eye. This zoonosis results from accidental ingestion of immature *Gnathostoma* larvae, a type of roundworm endemic to Southeast Asia and Latin America. The most common clinical presentation is dermatological—a cutaneous larva migrans [2]. However, intraocular gnathostomiasis is uncommon and can be localized to the anterior segment, retina, and vitreous cavity [12–19].

Myiasis is another parasitic infection that can affect the skin and the eye (ophthalmomyiasis). The causative organism is the immature larva of any fly of the order Diptera. Invasion of the globe is classified as ophthalmomyiasis interna anterior/posterior [20]. The clinical presentation of ophthalmomyiasis interna posterior (OIP) is very similar to that of intraocular gnathostomiasis, including uveitis (with or without eosinophilia) and self-limiting periorbital swelling [21]. For cases of OIP in which the larva is located within the subretinal space, photocoagulation may be used with minimal or no resultant inflammation [22, 23]. The subretinal pigmented tracks formed from larval migration within the subretinal space are pathognomonic for OIP [24].

Regarding the intraocular parasite of this patient, the SD-OCT findings are consistent with the findings on ophthalmoscopy. The internal reflectivity of the superior segments varies compared to that of the inferior segments. The hyper-reflectivity superiorly likely corresponds to a hyper-cellular esophagus represented as whitening grossly, and the hypo-reflectivity inferiorly corresponds to a relatively hypo-cellular intestinal tract represented as reddish brown coloration grossly. Closer observation of the inferior scans of the parasite reveals hyper-reflective lines connecting the outer lumen with the inner lumen. These hyper-reflective lines may represent lateral chords, and this configuration may be more suggestive of gnathostomiasis [25].

The high-resolution imaging of the worm and the acquisition of multiple images aligned along the body of the worm would be extremely difficult if not impossible with time-domain OCT.

SD-OCT with its high-speed scanning not only allows visualization of microscopic detail in vivo, but also allows volumetric, three-dimensional imaging. The Topcon 3D OCT-1000 (Topcon Medical Systems, Paramus, NJ) was able to render a three-dimensional representation of the worm in the vitreous. While patient history and visualization of the worm are helpful in diagnosing intraocular parasites, microscopic diagnosis is more definitive. SD-OCT may play an adjunctive role in identifying intraocular parasitic species prior to surgical removal and should be considered as part of the diagnostic imaging evaluation.

## Additional files

Additional file 1:
**Video 1.** SD-OCT video demonstrating two concentric circles within the vitreous above the retina. (WMV 6952 kb) Additional file 2:
**Video 2.** Three-dimensional SD-OCT video demonstrating the parasite within the vitreous above the retina. (WMV 6430 kb)

## Abbreviations

3D: three-dimensional
OIP: ophthalmomyiasis interna posterior
SD-OCT: spectral domain optical coherence tomography
